# Percutaneous endoscopic necrosectomy using a novel slim gastroscope with a large working channel for pancreatic walled-off necrosis

**DOI:** 10.1055/a-2839-9386

**Published:** 2026-04-22

**Authors:** Naoki Fujita, Hideki Kamada, Kiyoyuki Kobayashi, Manabu Yamada, Daisuke Namima, Hiroki Yamana, Hideki Kobara

**Affiliations:** 1 Department of Gastroenterology and Neurology, Faculty of Medicine 12850 Kagawa UniversityKagawaJapan

**Fig. 1:**
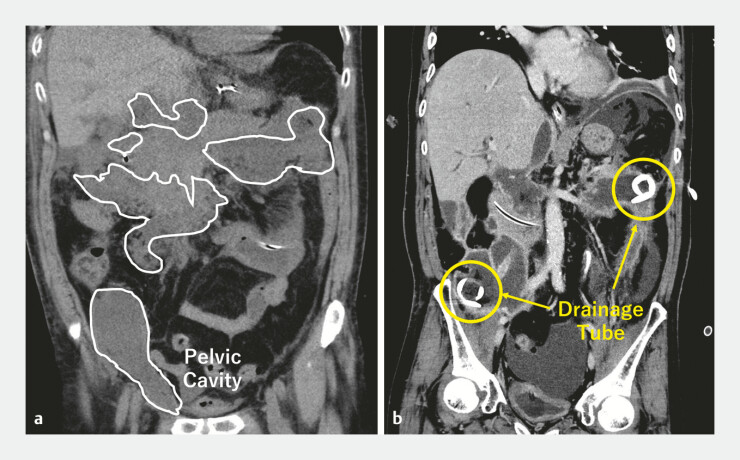
**a** A computed tomographic image demonstrating walled-off necrosis extending into the pelvic cavity. The area outlined in white indicates the walled-off necrosis. **b** As a safe puncture route to the pelvic walled-off necrosis was not available, and two 10.2-Fr percutaneous drainage catheters were placed (highlighted in yellow).

**Fig. 2:**
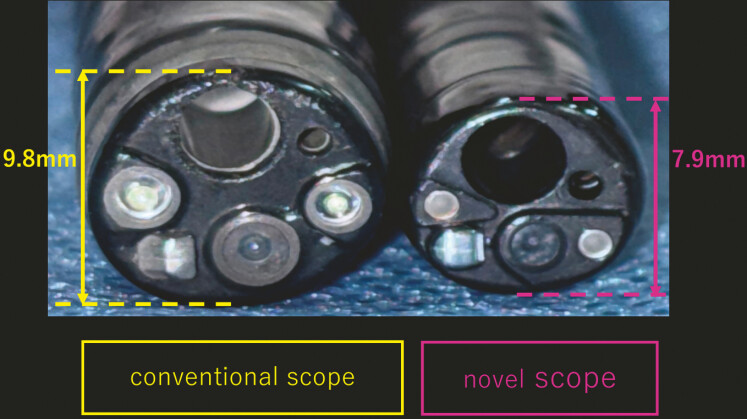
A slim gastroscope, which features a slim 7.9-mm outer diameter and a large 3.2-mm working channel. Additionally, the down-angle has been expanded from 120° to 160°, and the scope includes a waterjet function.

**Fig. 3:**
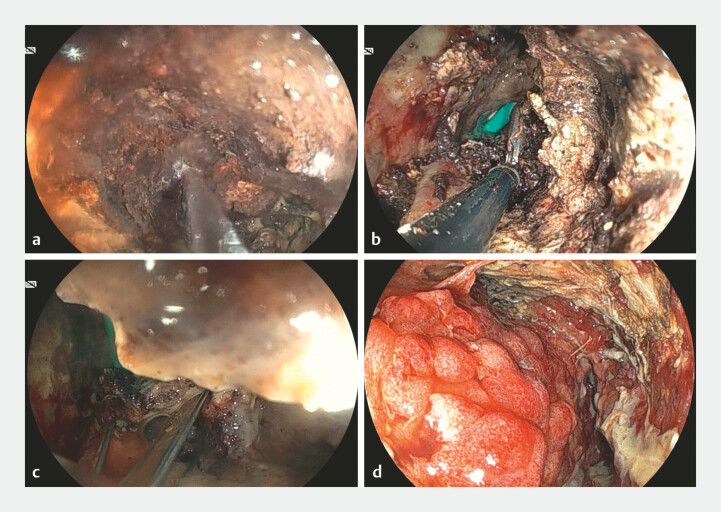
Endoscopic views during percutaneous endoscopic necrosectomy. **a** Jet irrigation provided powerful lavage, allowing efficient visualization and facilitating continuous necrosectomy. **b** Necrotic tissue was fragmented using alligator forceps. **c** A five-pronged forceps was used in a manner similar to panel **b** . **d** After further necrosectomy, no residual necrotic tissue was observed.

**Fig. 4:**
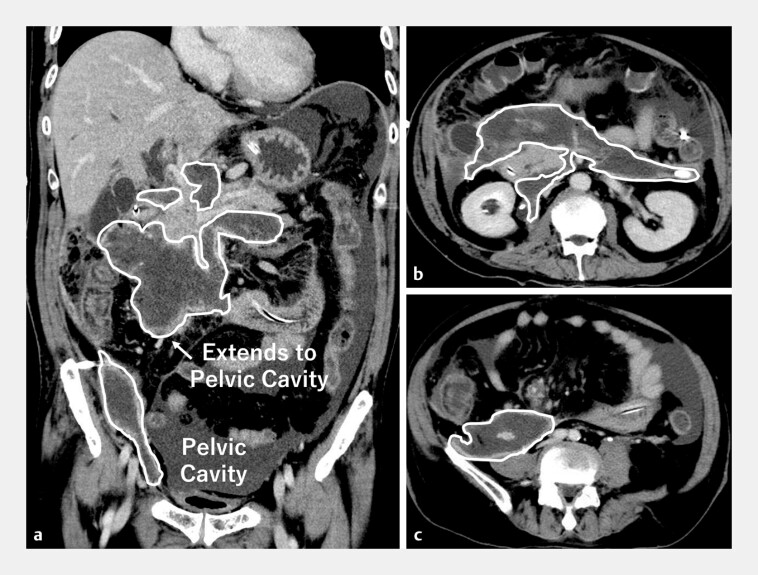
Computed tomographic images obtained before the initiation of percutaneous endoscopic necrosectomy. The walled-off necrosis, outlined in white, extended into the pelvic cavity. **a** A coronal view. **b** and **c** Axial views.

**Fig. 5:**
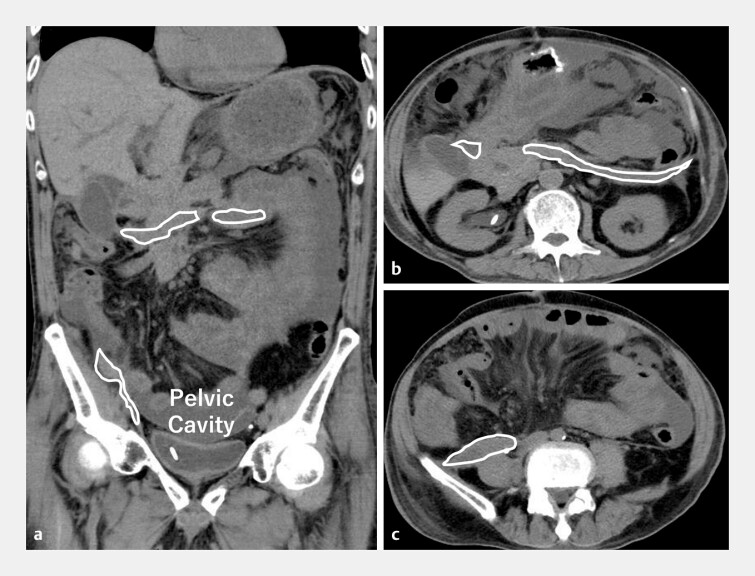
Computed tomographic images obtained after the completion of percutaneous endoscopic necrosectomy, shown at the same levels as in Fig. 4 . The walled-off necrosis, outlined in white, was markedly reduced in size. **a** A coronal view. **b** and **c** Axial views.

Percutaneous endoscopic necrosectomy (PEN) is a vital salvage technique for infected walled-off necrosis (WON) caused by severe pancreatitis when transluminal access is anatomically unsafe or when the cavity extends into the pelvic region 1 2 3 . A standard therapeutic gastroscope with a large outer diameter requires dilation of the access route to approximately 30–34 Fr 1 , resulting in delayed intervention. Meanwhile, an easy-access ultraslim endoscope cannot effectively evacuate the thick necrotic debris because of the narrow working channel 4 . Therefore, we investigated the newly developed 840TP gastroscope, which has a slim outer diameter of 7.9 mm, a large 3.2-mm working channel, and a working length of 1,100 mm 4 , making it suitable for PEN in anatomically complex situations. The small outer diameter allows early access through a relatively small percutaneous tract without extensive dilation, while preservation of a large working channel permits the use of standard necrosectomy devices, including alligator forceps, five-pronged grasping forceps, and snares, as well as effective suction of viscous necrotic debris. We report the case of a middle-aged man with infected WON extending to the pelvic cavity following acute necrotizing pancreatitis. Since a safe route for endoscopic ultrasound-guided drainage was unavailable, a percutaneous drainage tube was placed ( 5 ; Fig. 1 ). Despite the drainage, the infection persisted, necessitating a necrosectomy. Prior to the procedure, the percutaneous drainage tract was gradually upsized using drainage catheters; however, no additional tract dilation was performed at the time of endoscope insertion. At the time of the necrosectomy, the tract was occupied using an 18-Fr drainage tube and a 6-Fr catheter, creating a lumen of approximately 24 Fr. Because further dilation would have been required to introduce a standard therapeutic gastroscope and early necrosectomy was desirable given the patient’s severe inflammatory status and poor nutritional condition, a slim gastroscope was selected and successfully introduced into the WON via the existing percutaneous drainage route ( Video 1 ). The large working channel allowed the unhindered use of standard devices and effective suction of viscous necrotic debris ( Fig. 2 and Fig. 3 ). Furthermore, the widened 160° down-angulation and waterjet function facilitated efficient debridement, even in the difficult-to-reach pelvic extension. Complete necrosectomy was achieved over multiple sessions without intra-procedural or post-procedural adverse events. After the completion of treatment, the percutaneous drain was ultimately removed, and no repeat intervention was required. Although persistent external fistula formation is a potential long-term complication of percutaneous intervention, the fistula closed successfully in this case. Two months later, the WON was markedly diminished without additional interventions ( Fig. 4 and Fig. 5 ). PEN using a slim gastroscope, which enables early intervention through a small route and efficient suction, may be a promising treatment modality for complex WON, potentially leading to reduced procedural burdens and improved clinical outcomes.

Percutaneous endoscopic necrosectomy using a novel slim gastroscope for pancreatic walled-off necrosis.Video 1

Endoscopy_UCTN_Code_TTT_1AR_2AI
